# A New Antiseptic

**Published:** 1890-01

**Authors:** 


### A NEW ANTISEPTIC.

Sir Joseph Lister, the father of antiseptic surgery, has once more spoken to the world and has announced the completion of another step toward the perfection of that grand and successful system which was inaugurated by him twenty years ago. Starting out with carbolic acid as the basis of his antiseptic practice, he early recognized the objectionable features of this drug and substituted for it the bichloride of mercury as a more efficient and reliable germicide. About two years ago he in turn discarded the bichloride and adopted a combination of chloride of ammonium and bichloride of mercury, the "salalembroth" of the old chemists. Finding this so exceedingly soluble in the blood serum as to be practically useless, he experimented for a time with biniodide of mercury, but with little success on account of its irritating properties.

In the *British Medical Journal* of November 9, 1889, he announces that he has at length discovered the ideal antiseptic in the double cyanide of zine and mercury, a drug which is non-volatile, unirritating, and only slightly soluble in blood serum, and which in a strength of $\frac{1}{1200}$ is capable of keeping animal fluids permanently free from putrefaction. The only objection to this antiseptic lies in its deficiency of germicidal power, which Lister proposes to remedy by combining with it a weak solution $\frac{1}{1400}$ of bichloride, which is strong enough to be germicidal but too weak to be decidedly irritating. It remains to be seen whether this new antiseptic will prove to be the *ultima Thule* in this direction, or whether it will in turn give place to some more perfect preparation. Meanwhile we can but admire the indefatigable industry and perseverance of the illustrious investigator who has already revolutionized the art of surgery and accomplished results which might well satisfy the ambition of any ordinary man.

### ITEMS.

ATLANTA SOCIETY OF MEDICINE.—At the annual meeting for the election of officers of Atlanta Society of Medicine, the following officers were elected: Dr. Wm. Perrin Nicolson, President; Dr. Floyd W. McRae, Viçe-President; Dr. T. H. Huzza, Recording Secretary; Dr. E. van Goidtsnoven, Treasurer; Dr. N. O. Harris, Corresponding Secretary and Librarian; Drs. F. O. Stockton, L. P. Kennedy and E. V. Joye, Reporters.

Upon December 1, 1889, the Johns Hopkins Hospital, of Baltimore, commenced the publication of the *Hospital Bulletin*, which will contain announcements of courses of lectures, programmes of clinical and pathological study, details of hospital and dispensary practice, abstracts of papers and other proceedings of the Hospital Medical Society, reports of lectures and all other matters of general interest in connection with the work of the hospital. Nine numbers will be issued each year.

A NEW ENTERPRISE.—F. A. Davis, Medical and Scientific Publisher of Philadelphia, Pa., has established a branch office in Atlanta, and any medical or scientific work can be had at reduced rates by calling on J. M. Head, General Agent, No. 1, Kimball House.

---

LEICHTENSTERN (Koln), and H. CURSCHMANN (Leipzig). "On ileus and its treatment."—Congress for Internal Medicine, held from 15—18 April, 1889, at Wiesbaden.

(*Continued from Vol. X., page 466.*)

CURSCHMANN, in continuing the subject of ileus, limits himself to cases arising from sudden obstruction of the bowel, or, at least to ileus suddenly resulting from some chronic lesion, *e. g.:*

(1) Volvulus and intussusception.

(2) Constriction of the bowels by abnormal bands or cords, or passage of the bowels through abnormal or physiological openings.

(3) Barring of the bowel by foreign bodies and tumors.

(4) Compressions, deformities and strictures, with suddenly produced kinking displacement, or partial paralysis of the bowel.

In all these cases leading to ileus, Curschmann maintains that, in the early stages, a cure may be accomplished without operative procedures, and gives some teaching cases falling under his own observation. 105 cases (C. Goltdammer, Bülau) give thirty-five and one-fourth per cent. cures. Curschmann is by no means content with this, and thinks by using all the means in our power, this percentage might be raised, and he is candidly of opinion that early laparotomy has overstepped its bounds, which, in the future, will be more limited than now.

What are these bounds?

They consist in the difficulty of diagnosis, the condition of the patient, usually brought to hospital in a state of collapse unfavorable to operation, in the pulling out and packing in of the bowels to find the seat of strangulation. This being the case, he is in full accord with Schede's *dictum:* "A small or moderately large incision of the belly in the *linea alba*, or in a quarter as near as possible to the expected site of the obstruction, a quick sparing search after the lesion, without hauling out the intestines, and, if this is successful, the formation forthwith of an artificial anus by stitching the nearest extended coil of bowel to the belly wall."

In cases, though these are few in number, admitting a clear and early diagnosis, he advises early operation, although even then internal treatment is of great assistance to the surgeon.

The remedial measures he approves of are as follows: As soon as the first symptoms of ileus are apparent, the patient must at once be placed on absolute diet, as anything put into the bowel lying above the point of obstruction is simply a burden. For the thirst he recommends small pieces of ice flavored with brandy, and in some particular cases he has seen good effects produced by subcutaneous injections of solution of salt.

He lays much stress on the following:

(1) Trying to mitigate, at least in the first stages, the powerfully exaggerated, sometimes cramp-like, persistaltic motions above the point of obstruction.

(2) Using measures to lessen the over-filling and tension of the bowels.

(3) In certain cases employing known mechanical rules, having as their aim a direct removal of the obstacle or a bettering of the conditions causing it.

Finally, by carefully using means to cause peristalsis in the bowel beneath the obstruction, one may, perhaps, succeed in removing it.

As regards purging, he expresses himself decisively. *As soon as there is but a well-founded suspicion of incarceration of the bowel, away entirly with purging.* Purging can cause only mischief by increasing peristalsis.

In opium he recognizes an agent, almost without exception, favorable in its action, and advises its use always at the commencement. Often its results are magical. He gives it boldly every one or two hours, so as to amount to from one-half to one grain of pure opium in the twenty-four. With the commencement of paralysis the dosage must be regulated more carefully, or with interruptions.

Next to opium in palatable and curative power he places emptying and washing out of the stomach by the introduction of a tube, as practiced by Kussmaul. He has seen good results follow this, not only in the obstruction of the colon, but also in obstruction of the small intestine. Puncture of the bowel by an injection syringe he has used with good results more than once. In three cases, by this method, he accomplished a direct cure, and he never saw any bad results follow it; only he recommends care in its use, and disadvises it in the stage of paralysis or peritoneal irritation.

Frequent injection of fluid he does not approve of, as he does not think these can remove the obstruction by hydrostatic pressure.

Blowing air into the rectum, as practiced by Ziemssen, Runeberg and others, he considers far preferable. The method is easily carried out and entirely free from risk. He has had some speedy cures by it where the site of the obstruction lay in the large intestine.

In the discussion that followed many recognized authorities took part. What follows is mainly a short *résume* of the opinions that differ from those of Leichtenstern and Curschmann.

Jürgensen (Tubingen) gives a case in which he blew air into the rectum with this result that the patient died in a few seconds from sudden general emphysema. There was no perforation of the bowel, but the cause of obstruction being a cancer, the strong pressure had forced the bowel asunder and let the air into the retro-peritoneal connective tissue, causing a double pneumothorax, resulting in death.

Rosenbach (Breslau) describes a burgundy red reaction, produced by slowly dropping acid nitrate of potash into urine kept at the boil. Besides the deep red the appearance of a blue-violet foam is characteristic. The reaction means grave tissue change, and accordingly is of value in determining the more or less serious nature of the obstruction.

Nothnagel (Vienna) has never seen vomiting of *formed fæcal masses*. Such masses are almost invariably formed behind the ileo cœcal valve, and vomiting these would seem an impossibility. Any masses he has seen have been milk clots stained outside with bile.

Ziemssen (Munich) considers blowing air into the rectum by no means dangerous, and in his many cases he has had no bad results. A communication between stomach and transverse colon might easily produce vomiting of fæcal masses and lead to a wrong diagnosis. Opium and morphia he would introduce into the rectum or give subcutaneously, so that it might be entirely absorbed. He approves of puncture and of washing out the stomach.

Leube (Wurzburg) asks two questions. When *dare* one and when *must* one operate? He answers the first thus: When opium has been given in large doses and a purge [he still clings to this], and the stomach washed out [he has not seen much good from that], then, even if the pulse be good, one dare operate. And the second thus: When the pulse shows, be it but the faintest sign of failing, then it is our duty to operate.

Baumgärtner (Baden) approves of early operation, even in uncertain diagnosis.

Nothnagel (Vienna) deplores the uncertainty that still hampers the treatment, and our absence of knowledge about the action of the remedies employed. He strongly objects to purging. Opium he as strongly approves of, although he cannot conjecture how it acts. [It is worthy of remark that throughout the whole discussion, as given in the German abstract, although artificial anus is mentioned, there is no reference to resection of the bowel, or Senn's (Milwaukee) remarkable experiments on animals, and the bold and successful abdominal surgery of Lawson Tait is altogether lost sight of. Baumgärtner, of Baden, is the only one who advises bold and early surgical treatment.]—*W. A. Stewart in Medical Chronicle.*

BOURGET. "The chemical changes in the gastric juice."—*Revue Médicale de la Suisse Romande*, December 20, 1888.

Dr. Bourget, in this paper, after criticising the theories of Bouchard and Glenard, proceeds to give the result of his experiments with regard to the action of certain substances upon the contents of the stomach. Salol only undergoes change after passing the pylorus and coming in contact with an alkali. Dr. Bourget found that on taking 2 per cent. HCl with a meal salol was discovered in the urine 1¼ to 1½ hours afterwards, while if he partook of fruit and vegetables the time which elapsed was reduced to from a quarter to half an hour. He argues from this that conclusions drawn from the use of salol are subject to great error. Dr. Bourget objects to the common plan of prescribing pepsin in disease of the stomach. He says that in sixty-three cases, after analysis of the contents of the stomach, sufficient pepsin was present for the purposes of digestion. In fifteen cases of carcinoma pepsin was never deficient in quantity. This was also the case in forty cases of chronic and subacute gastritis. In sixteen cases of carcinoma Dr. Bourget never found full HCl in the stomach. In seven cases of ulcer of the stomach the quantity of HCl was great, but no pyrosis was present. In thirty-six cases of gastritis under observation HCl was absent, only to return with return of health. When HCl is absent lactic acid is present. In a case of chronic gastritis Dr. Bourget found 1.60 per cent. of lactic acid and no HCl. The lactic acid being the product of micro-organisms in the stomach, the process of their development is checked by HCl. Bicarbonate of soda is useful, but brings no permanent benefit. Instead of using these drugs, he says, "Prevent the formation of lactic acid by the administration of HCl." From these observations he concludes (1) that pepsin is nearly always useless in the treatment of these diseases; (2) that pyrosis ought to be combated not by alkalies, but by HCl; (3) and that alkalies should never be given until the process of digestion is completed.—*James McNaught in Medical Chronicle.*

KENDAL FRANKS. "Report on massage."—*Dublin Journal of Medical Science*, September, 1889.

Since massage was introduced into this country, by the appearance of Dr. Weir Mitchell's book on "Fat, Blood, and how to make them," attention has become more and more fastened upon this therapeutic agent, not only as the remedy *par excellence* in neurasthenia, but also for various local ailments and constitutional diseases. Much literature has now accumulated on this subject and the report of Dr. Kendal forms a good *résumé* of these various publications.

The manipulations employed in massage are usually four, viz.: (1) *Effleurage*, applied to all stroking forms of movement, light movements, free from pressure, which may be carried on with the tip of one finger, or the tips of all the fingers, or with the backs, sides, or palms of the hands. Effleurage manipulations are particularly applicable to the head, neck, and other extremities. (2) *Pétrissage* means pinching, kneading, and working into the deeper structures by bringing, or rather massing, them together. This is, perhaps, the most important of all the manipulations. (3) *Tapotement* is a kind of percussion which may be made with the tips of the fingers, palms of the hand, borders of the hand, or with the ulnar side of the loosely closed hand. Especially applicable to the back, chest, and abdomen, and to the trunk of the body generally. (4) *Friction or massage à frictions* has nothing to do with what we ordinarily term friction, but consists of two distinct modes of procedure, thus described by Beuster: "The finger tips of one hand, held at right angles to the axis of the limb, rub across and across in narrow ellipses, while the fingers of the other hand stroke parallel to the axis of the limb." This process is chiefly employed in the treatment of affections of the joints.

These chief divisions are capable of indefinite subdivisions to suit the particular fancies of the operator.

The physiological effects of massage are various. In a paralyzed limb of a child suffering from anterior poliomyelitis, massage caused "increased temperature and change of color from lividity to bright red, these changes being, of course, associated with increased oxidation, increased molecular activity, and increased sensibility and metabolism." In this case also, after massage, there was a distinct increase in the excitability of the muscles to galvanism—eleven milliampères current strength before massage, and 5 milliampères after massage, being required to cause muscular response, the temperature of the limb being raised 25° F. by masséing.

Dr. Schrieber thus sums up the physiological effects of massage:

"To cause an increased flow of blood to muscles and soft parts, increasing thereby the circulation, and removing accumulations of waste tissue whose retention causes various disturbances of function.

"To strengthen muscle fibres, and by getting up molecular vibrations to induce changes not only on the muscle and nerve fibres but perhaps even in the nerve centres themselves.

"To cause the resorption of exudations, transudations, and infiltrations in such organs as are accessible. To effect the separation of adhesions in tendon sheaths and in joints without recourse to the knife. To remove, by grinding away, intra-arthritic vegetations.

"To increase by passive and active exercise of all the muscles the oxidizing powers of the blood, in this way correcting disturbances of its composition and stimulating all the vegetative processes.

"To reduce the congestion of such internal organs as the brain, lungs, intestines, uterus, kidneys, etc., by increasing the flow of blood to the muscles.

"To stimulate directly the sympathetic nervous system, thus increasing secretion and reflexly the activity of unstriped muscle fibre, and so relieving various functional derangements.

"By systematic exercise (Health Gymnastics) to educate morbidly affected muscles, to convert abnormal into normal actions, and to suppress useless movements.

The number of diseases, both local and general, which have yielded to massage properly performed has in late years very much increased.

This method of treatment, combined with absolute rest, overfeeding, isolation, and electricity, is especially useful in all affections which can be directly or indirectly ascribed to hysteria, or to a neurasthenic condition. In paralytic affections massage has been repeatedly tried, and with varied success, hysterical cases and other functional conditions being most successful; but benefit is also claimed in paralysis due to organic lesions of the cord, especially in infantile paralysis, pseudo-hypertrophic paralysis, facial paralysis, wasting palsy, and allied affections. Several cases of cure in chronic myelitis are on record; also of benefit in Landry's paralysis, and in allaying the acute pains of locomotor ataxy. In writer's cramp much success has been obtained by a modified system of "rubbing" and gymnastics. In some cases of paralysis—viz., those due to lateral sclerosis—massage has apparently done harm.

Localized massage is frequently useful in localized neuralgia, especially sciatica, and also in megrim and congestive headache.

Massage has become a recognized method of treatment in many joint affections, particularly sprains, whether recent or of old standing. It has also been used in cases of synovitis, both rheumatic and traumatic.

In June, 1887, Dr. Berne advocated the treatment of certain *fractures* by massage, the most suitable being fractures of the radius and fibula, the unbroken ulna or tibia acting as a natural splint. Other cases are fractures near to and involving joints, and also fractures of the olecranon and of the patella.—*Robert B. Wild in Medical Chronicle.*

---

THE WORK ALREADY DONE IN THE DIRECTION OF STANDARDIZING FLUID PREPARATIONS.—The first and most notable advance made in the direction of supplying standardized preparations not open to the dangers of the existing Pharmacopœial processes for fluid extracts, was by Messrs. Parke, Davis & Co., who introduced, in 1883, a class of assayed preparations which were entitled Normal Liquids. The standard decided upon for these fluids was the result of long experience in the collection, purchase, examination and analysis of crude drugs, with a determination of the amount and character of their active principles. The reliability of normal liquids soon led to their large consumption, and the medical profession have evinced their preference for them to such an extent as to make them now an established and popular method of exhibiting the toxic and narcotic drugs.

Normal liquids may be defined to be concentrated tinctures, the methods of manufacture of which serve as models for imitation. They represent more closely than fluid extracts made by the present Pharmacopœial methods the average standard strength of crude drugs. The simplest explanation of their nature would probably be to regard them as fluid extracts adjusted by assay to a fixed standard of strength which makes them absolutely uniform in composition and therapeutic action.

The favor with which normal liquids, and assayed products generally, have been received by representative men of the medical profession has led us to believe that the best interests of pharmacy will not be served unless these or like preparations are officially recognized. For concentrated tinctures of a definite strength, the name "normal liquids" appears to be happily chosen, as it implies a definite standard of strength. The list should embrace preparations of the more potent crude drugs, 1 Ccm. representing 1 gramme of drug of standard strength.

It does not seem to us from a careful review of all efforts made in this direction that any have met with equal acceptance, or merit as much appreciation. Whatever may prove to be the decision of the committee as to making such assayed preparations official, there can never be any question as to whom the honor of their actual practical introduction is due.

As the time approaches when the revision is to take place (and in the minds of thinking men the standardization of fluid extracts is now an accepted fact), there will no doubt be many competitors for this honor who may claim, by reason of a mushroom-like growth in the field of this new departure, official recognition for scientific work.

It will be necessary on the part of the Committee of Revision, therefore, to carefully investigate the claims in this direction, and when awarding the credit for such work to see that they do not place the laurels upon the wrong brow.

Unsupported and disinterested scientific labor, no matter from what source, should always be welcomed with the endorsement of scientific men, and we sincerely trust that the efforts made in this direction by those deserving it, will receive full appreciation at the hands of the compilers of the forthcoming Pharmacopœia. —*Reprinted from Medical Age, Nov. 25, 1889.*

